# GPGMH, a New Fixed Timed-AI Synchronization Regimen for Swamp and River Crossbred Buffaloes *(Bubalus bubalis)*

**DOI:** 10.3389/fvets.2021.646247

**Published:** 2021-09-06

**Authors:** Adili Abulaiti, Hadeel S. El-Qaliouby, Halla E. K. El Bahgy, Zahid Naseer, Zulfiqar Ahmed, Guohua Hua, Liguo Yang

**Affiliations:** ^1^Key Laboratory of Animal Genetics, Breeding and Reproduction, Ministry of Education, College of Animal Science and Technology, Huazhong Agricultural University, Wuhan, China; ^2^Department of Animal Wealth Development, Faculty of Veterinary Medicine, Benha University, Toukh, Egypt; ^3^Department of Veterinary Hygiene and Management, Faculty of Veterinary Medicine, Benha University, Toukh, Egypt; ^4^Faculty of Veterinary and Animal Sciences, Pir Mehr Ali Shah Arid Agriculture University, Rawalpindi, Pakistan; ^5^International Joint Research Centre for Animal Genetics, Breeding and Reproduction, College of Animal Science and Technology, Huazhong Agricultural University, Wuhan, China; ^6^Hubei Province's Engineering Research Centre in Buffalo Breeding and Products, College of Animal Science and Technology, Huazhong Agricultural University, Wuhan, China

**Keywords:** GPGMH, estrus synchronization, mifepristone, TAI, crossbred buffalo

## Abstract

The crossbreeding of Swamp and River type buffalo breeds is practiced for the improvement of milk yield and reproductive performance in swamp buffalo herds. This study aimed to modify the Ovsynch synchronization protocol (GPG) and improve the fixed-timed artificial insemination (FTAI) for better reproductive performance of crossbred buffaloes. Comparison of four conventional synchronization protocols [pregnant mare gonadotropin-prostaglandin F_2_α-gonadotropin-releasing hormone (PmPG), gonadotropin-releasing hormone-prostaglandin F_2_α-gonadotropin-releasing hormone (GPG), prostaglandin F_2_α-gonadotropin-releasing hormone-prostaglandin F_2_α-estradiol benzoate (PGPE), and progesterone-pregnant mare gonadotropin-prostaglandin F_2_α-gonadotropin-releasing hormone (P_4_PmPG)] in crossbred buffaloes showed that the GPG protocol treated buffaloes displayed higher (*P* < 0.05) estrus response with an increasing tendency in ovulation (84.6%) and pregnancy rates (30.8%) than PmPG, PGPE, and P_4_PmPG treated buffaloes. Buffaloes treated with a dose of 0.4 (mg/kg) mifepristone combined with GPG, exhibited higher (*P* < 0.05) estrous response (82.4%), ovulation (94.1%), and pregnancy (47.1%) rates compared with other doses (0, 0.3, or 0.5 mg/kg) groups. Injection of mifepristone along second GnRH injection in buffaloes improved (*P* < 0.05) pregnancy rate (35.3%) when compared to before or after the second GnRH of GPG protocol. Single AI after 24 h of mifepristone or second GnRH injection seems the best time to enhance the pregnancy rates in buffaloes compared to double or other single AI times in the modified GPGMH protocol. In comparison, GPGMH reduced the follicular cyst incidence (*P* < 0.05) with increasing ovulation (*P* > 0.05) and pregnancy rates (*P* > 0.05) than the P_4_GPG and GPG protocols in crossbred buffaloes. The current study supported that new synchronization protocol (modified of GPG protocol; GPGMH) by the inclusion of mifepristone (with a dose of 0.4 mg/kg along second GnRH), AI after 24 h of mifepristone or second GnRH, and human chorionic gonadotropin (hCG at day 5 of AI) enhance the ovulation and pregnancy rates in crossbred buffaloes.

## Introduction

The buffalo is the second largest species of dairy livestock in the world. China has the third highest population of buffaloes in the world, following India and Pakistan. However, Chinese domestic buffaloes belong to swamp breeds, which have lower milk production compared to riverine breeds (Murrah, Nili-Ravi, and Mediterranean buffalo) (1). Crossbreeding of swamp with river buffaloes has been an effective strategy to improve milk yield (2). Crossbreeding of swamp (2*n* = 48) with river buffaloes (2*n* = 50) resulted in a hybrid progeny (49 chromosomes) (3) and crossbred progeny of buffaloes has relatively lower fertility than pure breeds. In general, compared with cattle, buffalo exhibit higher reproductive problems such as seasonal breeding, delayed puberty, longer post-partum intervals, and silent heat (4). These factors make it difficult to apply synchronization protocols vigorously, as practiced in cows. In this scenario, the crossbred progeny of buffaloes needs greater attention for reproductive management.

Synchronization techniques involve practical benefits, such as bringing a large percentage of buffaloes into estrus at a predetermined time and FTAI during the breeding and non-breeding seasons (5). FTAI is considered as an effective mode of breeding to improve bovine fertility (6). Similar to cows, several FTAI protocols were tested in buffaloes such as pregnant mare serum gonadotropin (PMSG)-Prostaglandin F_2_α (PGF_2_α)-Gonadotropin-releasing hormone (GnRH)-[PmPG] (7), GnRH-PGF_2_α-GnRH-[GPG] (8), PGF_2_α-GnRH-PG-Estradiol benzoate (EB)-[PGPE] (9), and Control internal drug release-CIDR-PMSG-PGF_2_α-GnRH-[P_4_PmPG] (10). The most commonly used estrus synchronization and FTAI protocol in lactating buffaloes is the Ovsynch program, previously GPG (11), which resulted in around 50% conception rate during the breeding season in buffaloes (12).

Follicular cyst and silent heat are the most frequent reproductive disorders in buffaloes, leading to infertility and extended calving intervals (13). Mifepristone is a kind of progesterone (P_4_) receptor antagonist, which works as an antiprogestogen by blocking the progesterone receptors, in turn rapidly reducing the P_4_ level, and further promoting luteinizing hormone (LH) surge for ovulation (9). Inclusion of mifepristone in GPG protocol prior to AI seems a useful strategy to lower the P_4_ level for better expression of heat signs and reduction of follicular incidence, particularly in buffaloes. Human chorionic gonadotropin (hCG) is a hormone produced by the human placenta after embryo implantation, which interacts with its receptors in the ovary and promotes the corpus luteum (CL) to secrete more P_4_ during the first trimester for pregnancy maintenance (14). The incorporation of hCG in GPG protocol was hypothesized to increase the application of GPG protocol in buffaloes on a broader aspect. Therefore, the addition of mifepristone (before AI for reducing P_4_ level) and hCG (the fifth day after AI for promoting CL activity for P_4_ synthesis) to conventional TAI regimen will efficiently improve the application of AI and fertility in crossbred buffaloes.

## Materials and Methods

### Care and Use of Animals

Use of animals and all experimental procedures were performed following the guidelines of the Committee of Animal Research Institute, Huazhong Agricultural University, China, and the Ethical Committee of the Hubei Research Center of Experimental Animals (Approval ID: SCXK (Hubei) 20080005).

### Experimental Animals

The present study was conducted in Hubei province (37.8957° N, 114.9042° E), China during breeding and non-breeding seasons (September 13, 2017-December 30, 2018). A total of 433 crossbred buffaloes (Mediterranean × Murrah or Nili Ravi × Jianghan), varying from 3 to 6 years old (first to third lactations), with moderate body weight (636.07 ± 430.5 kg) and body condition score (2.5 to 3 points; 1-5 scale), were selected from a buffalo farm in Hubei, China (Hubei Jinniu Co., Ltd.). The buffaloes were ~90–100 days in milk, and calves were weaned after 4 weeks of calving. All the animals were cyclic, reproductively sound with good general health, and physical condition was nearly the same size in each group. The cyclicity status of buffaloes was based on the regular estrous cycle and observation of follicular and CL development through regular ultrasonography examinations. The stall feeding system was in practice and buffaloes were kept lose in a head-to-head feeding system. The animals were fed on a total mixed ration (TMR) consisting of forage (corn silage, peanut vine, rice straw) with concentrate (corn; 38%, soybean meal; 16%, linen; 6.0%, cottonseed cake; 6%,corn meal; 17.5%,vinasse; 10%,little su; 0.5% and premixed material; 6%). Fresh and clean water was accessible 24 h to each animal. Milking was practiced using a milking machine twice a day (6:00 and 18:00).

### Experiment 1

**Figure 1:**
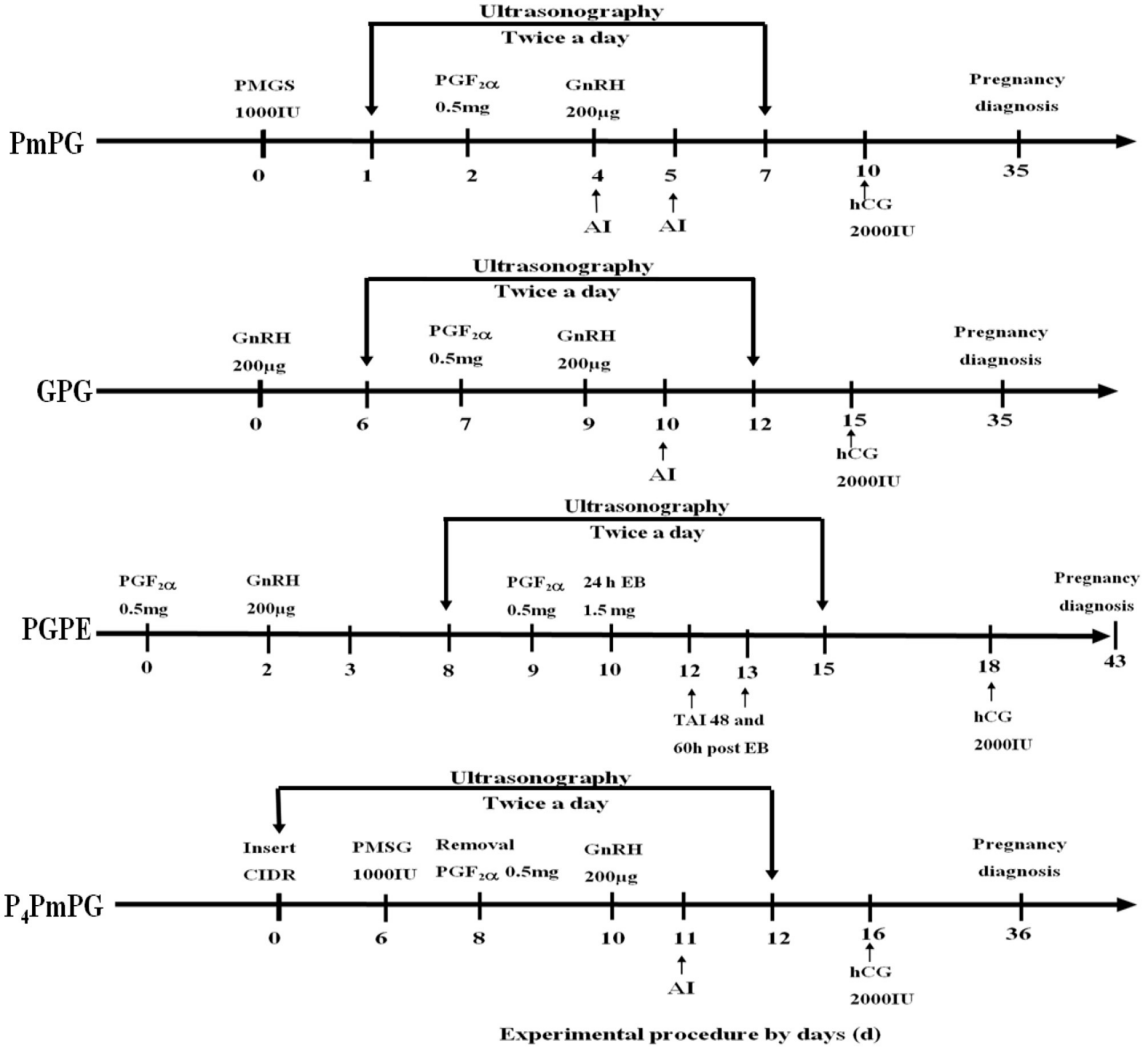
Schematic diagram of four conventional synchronization-TAI protocols (P_4_PmPG, GPG, PGPE, and PmPG) used in crossbred buffaloes during breeding season. The two downward vertical arrows in the window present the ultrasonographic observations of buffaloes synchronized through P_4_PmPG, GPG, PGPE, and PmPG protocols.

Experiment 1 was conducted to select an optimum FTAI synchronization protocol for crossbred buffaloes. A total of 94 animals were randomly divided into four groups: PmPG, GPG, PGPE, and P_4_PmPG (Figure 1). PmPG group (*n* = 28) of buffaloes was treated with PMSG [1,000 IU, intramuscularly (I.M.) Ningbo Sansheng Pharmaceutical Ltd, China] on day 0, PGF_2_α (0.5 mg, I.M., Ningbo Sansheng Pharmaceutical Ltd, China) and GnRH (200 ug, I.M., Ningbo Sansheng Pharmaceutical Ltd, China) were injected on day 2 and day 4 subsequently (7). An injection of hCG (2,000 IU, IM, Ningbo Sansheng Pharmaceutical Ltd, China) was given on day 10 of the protocol. Buffaloes were scanned for follicular dynamics (follicles diameter recordings, and ovulation; sudden disappearance of Graffian follicle on subsequent scan) twice (7:00 and 19:00) a day starting from day 1 to 7 of the protocol through ultrasound machine (WED-9618-v, equipped with LV2-3/6.5 MHz rectal probe, Shenzhen Well.D Medical Electronics Co., Ltd., Guangdong, China). Buffaloes were observed for estrus signs visually (vulvar edema, vaginal mucus discharge, pinkish vaginal mucosa, and bellowing) and rectal exam (uterine tone and presence of large follicle >9 mm on either ovary through ultrasound) regularly for recording the silent estrus incidence. AI was done on days 4 and 5 of the protocol using cryopreserved semen (Semen Cryopreservation Station, Hubei, China), and pregnancy diagnosis was carried out on day 35 of the protocol through ultrasound rectally.

The GPG group (*n* = 26) was initially treated with GnRH (200 ug) on day 0, later an injection of PGF_2_α (0.5 mg) on day 7 and GnRH on day 9 was administered (8). In this group, follicular dynamics were monitored ultrasonographically between day 6 and 12, observed for estrus, and AI was done on day 10 (24 h after the second GnRH of protocol). A dose of hCG hormone (2,000 IU) was injected into each buffalo on day 15 and diagnosed for pregnancy on day 35 of the protocol.

In the PGPE group (*n* = 26), the buffaloes were synchronized by adopting the procedure, first PGF_2_α (0.5 mg) on day 0, GnRH (200 ug) on day 2, second PGF_2_α (0.5 mg) on day 9 and Estradiol benzoate (1.5 mg, I.M. Ningbo Sansheng Pharmaceutical Ltd, China) on day 10 (9, 15). The PGPE treated buffalo were monitored for follicular development (between days 8 and 15, twice a day through ultrasound), estrus expression, and each buffalo was inseminated twice at 48 and 60 h after EB treatment. On day 18 of the protocol, each buffalo was treated with hCG (2,000 IU) and further submitted to pregnancy estimation on day 43 of the protocol.

The P_4_PmPG treated buffaloes (*n* = 14) were initially treated with CIDR (Ningbo Sansheng Pharmaceutical Ltd, China) for 8 days and were given PGF_2_α at the time of CIDR removal. The PMSG and GnRH were injected to each buffalo of the group on days 6 and 10 of the protocol, respectively (10). The ovaries of submitted buffaloes were observed for follicular dynamics from CIDR insertion to day 12 of protocol and estrus signs were recorded. The buffaloes were inseminated on day 10 and treated for hCG injection on day 16 of the protocol. The pregnancy diagnosis was performed on day 36 of the protocol.

### Experiment 2

The best-evolved protocol, GPG, from experiment 1 was further tested to optimize a dose of mifepristone for crossbred buffaloes. For this purpose, a total of 71 buffaloes were divided into four groups and treated with four different doses of mifepristone (Hubei Yun Cheng Sai Technology, China): 0 (GPG, 0 mg/kg *n* = 20), 0.3 (0. 3mg/kg *n* = 17), 0.4 (0.4 mg/kg; *n* = 17), and 0.5 (0.5 mg/kg; *n* = 17), respectively. Initially, the buffaloes were synchronized through GPG protocol (first GnRH on day 0, PGF_2_α on day 7, and second GnRH on day 9). The injection of mifepristone (0, 0.3, 0.4, or 0.5 mg/kg to the respective group) was given simultaneously to the second GnRH injection and inseminated 24 h after the treatment. The buffaloes were scanned for ovarian dynamics starting from day 6 to 12 of protocol and observed for estrus signs. An injection of hCG (2,000 IU) was given on the fifth day after AI and diagnosed for pregnancy at day 35 of the protocol.

### Experiment 3

Experiment 3 was conducted to optimize the injection time of mifepristone of the best chosen dose (0.4 mg/kg at the time of the second GnRH) from experiment 2 in GPG based protocol for crossbred buffaloes. In this experiment, 52 included buffaloes were synchronized through GPG based protocol (first GnRH on day 0, PGF_2_α on day 7, and second GnRH on day 9), and randomly divided the buffaloes into three groups. The first group (*n* = 17) of buffaloes was treated with mifepristone (0.4 mg/kg) at the same time as the second GnRH injection, and the second group (*n* = 18) received mifepristone (0.4 mg/kg) 4 h before the second GnRH injection. The third group (*n* = 17) was treated with mifepristone (0.4 mg/kg) 4 h after the second GnRH of the protocol. Buffaloes were monitored using an ovarian picture (twice daily using ultrasound starting from day 6 to 12 of protocol) and estrus display. Later, all treatment groups, after 24 h of the second GnRH were undergone for FTAI. Following, hCG (2,000 IU) was given to all groups on the fifth day of FTAI. Pregnancy diagnosis was made using an ultrasound machine on day 35 of the protocol.

### Experiment 4

Experiment 4 was designed to optimize the AI time following the previously optimized GPGMH treatment. In this experiment, we assessed the optimum time of AI (20, 24, or 28 h) after second GnRH and frequency of AI (single vs. double) following optimized GPGMH (first GnRH on day 0, PGF_2_α on day 7, second GnRH, and mifepristone on day 9 and hCG 5 days post-AI) protocol from experiment 1, 2, and 3. About 100 buffaloes were divided into five groups. In the first three groups, single-time AI was performed at 20 h (*n* = 16), 24 h (*n* = 16), and 28 h (*n* = 17) after mifepristone or second GnRH injection, respectively. To compare the timing of double AI (20 and 28 h vs. 16 and 26 h) after mifepristone or second GnRH injection, one group of buffaloes (*n* = 25) was inseminated twice at 20 and 28 h after mifepristone or second GnRH injection, whereas, buffaloes (*n* = 26) in the second group were inseminated double time at 16 and 26 h following mifepristone or second GnRH injection.

### Experiment 5

**Figure 2:**
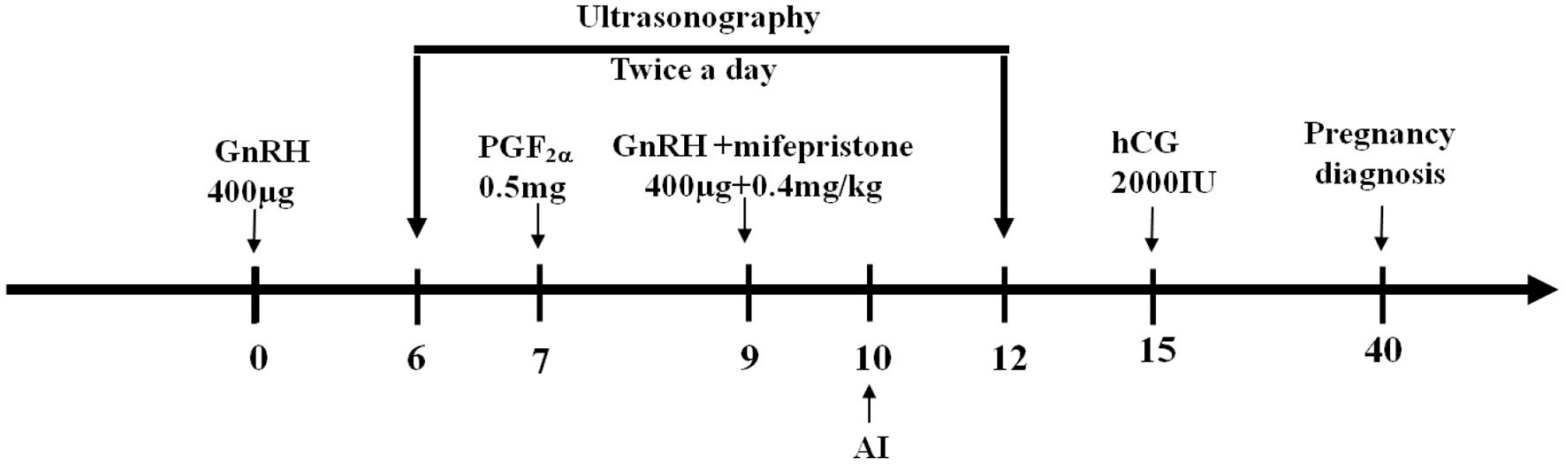
Schematic diagram of GPGMH method. Crossbred buffaloes have received an intramuscular injection of 400 μg GnRH on day 0, followed by an injection of 0.5 mg PGF2α on day 7, the second injection of 400 μg GnRH and one injection of 0.4 mg/kg mifepristone on day 9, artificial insemination on day 10, an injection of 2,000 IU hCG on day 15, and a pregnancy diagnoses on day 40 of protocol.

To observe the effect of P_4_ and its antagonist in FTAI programs, GPGMH, P_4_GPG, and GPG protocols were compared for crossbred buffaloes. A total of 116 buffaloes were treated with GPGMH (*n* = 45; first GnRH on day 0, PGF_2_α on day 7, second GnRH, and mifepristone on day 9, AI after 24 h of second GnRH and hCG 5 days post-AI; Figure 2), P_4_GPG (*n* = 41; first GnRH and CIDR insertion on day 0, PGF_2_α and CIDR removal on day 7, second GnRH on day 9, AI 24 h after second GnRH and hCG 5 days post-AI) and GPG (*n* = 30; first GnRH on day 0, PGF_2_α on day 7, second GnRH on day 9, AI 24 h after second GnRH and hCG 5 days post-AI). All the treatment groups were monitored for follicular dynamics through ultrasound, estrus display, and pregnancy diagnosis at specific moments (Figures 1, 2) (16).

### Statistical Analysis

**Figure 3:**
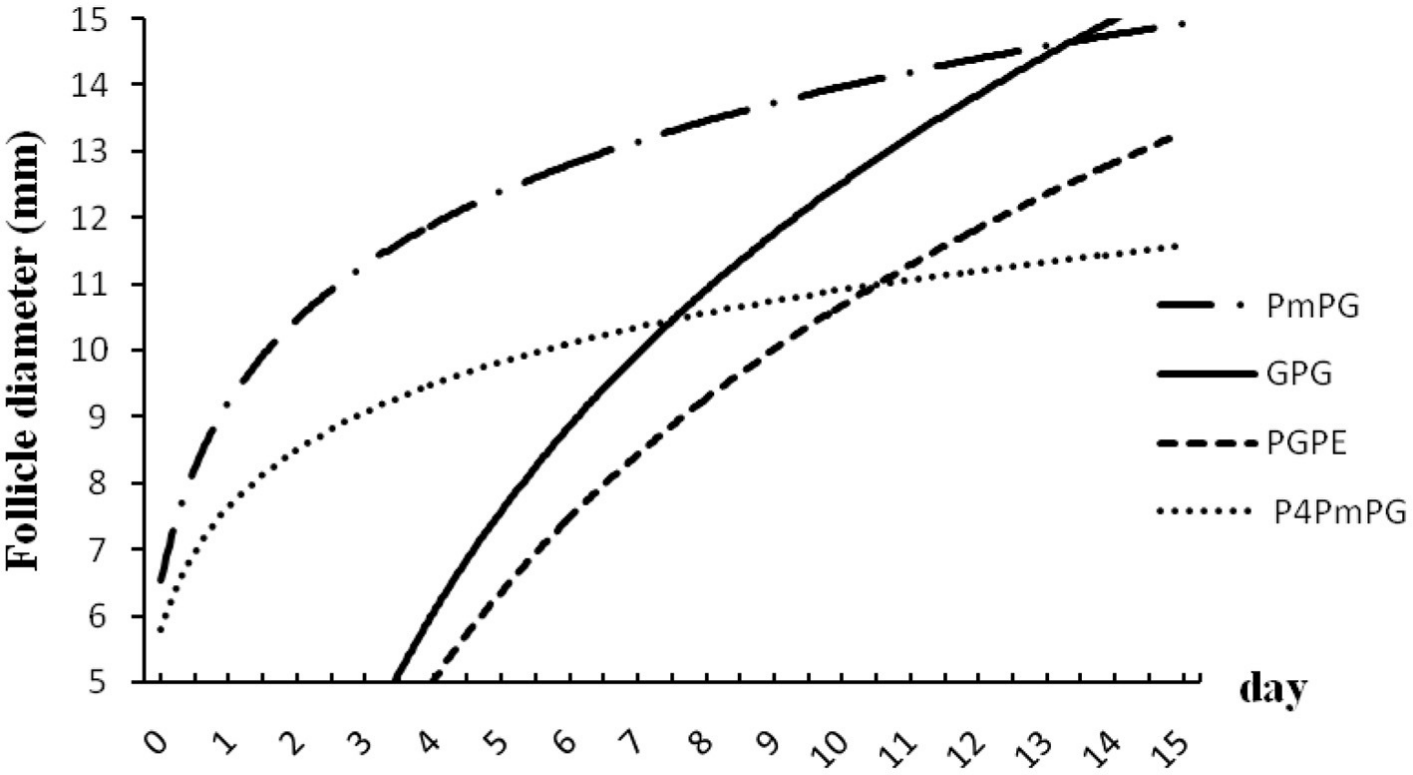
Follicular dynamics in crossbred buffaloes subjected to different TAI synchronized protocol (PmPG, GPG, PGPE, and P4PmPG). Values in both X- and Y-coordinates were transformed in logarithm to compare the different protocols.

**Figure 4:**
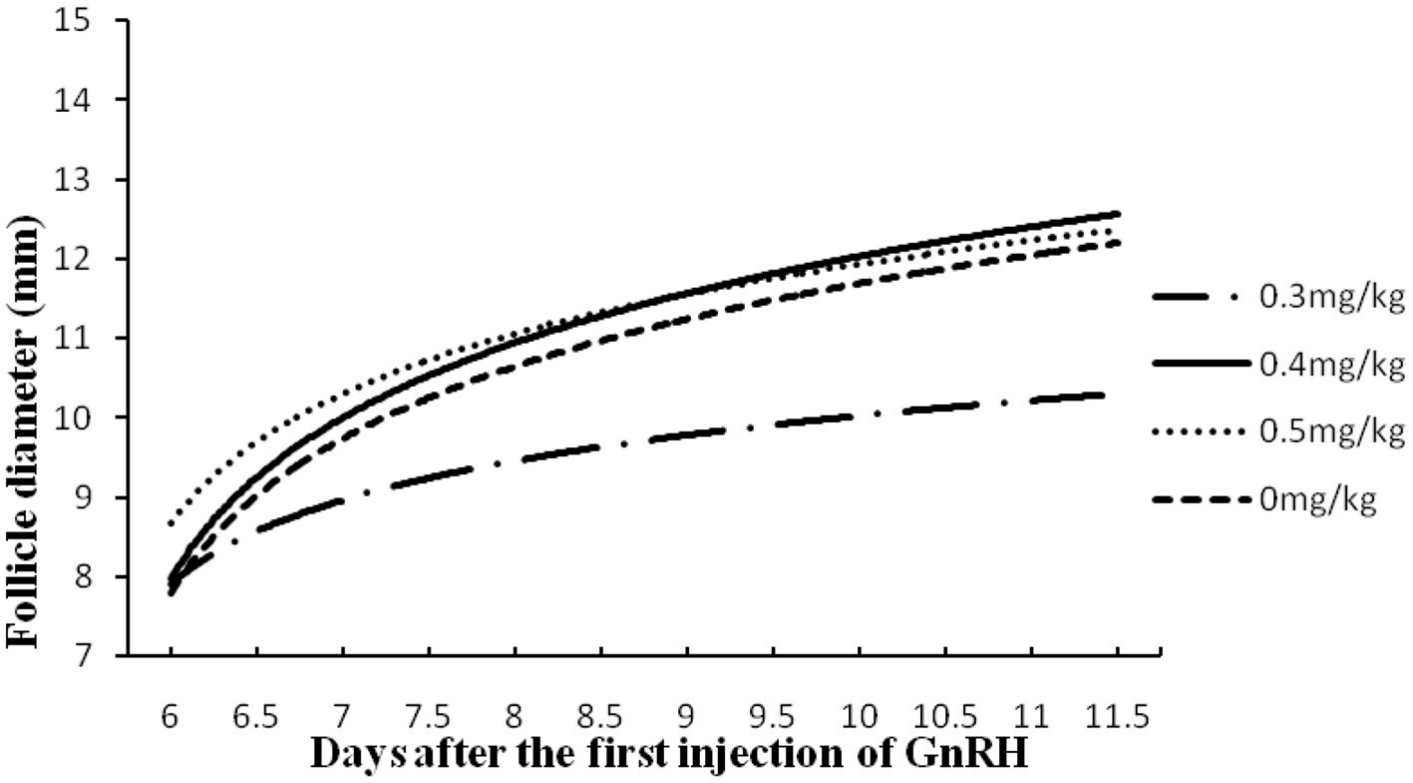
Follicular development in buffaloes synchronized by GPGMH with different doses of mifepristone (0, 0.3, 0.4 and 0.5 mg/kg). Values in both X- and Y-coordinates were transformed in logarithm for comparison of groups.

Data of follicle diameters given in Figures 3, 4 were firstly transformed in logarithm. ANOVA was used to analyze the ovulatory follicle diameter, and the growth of dominant follicles among different groups was expressed as mean ± standard error (SEM). The chi-square test was used to compare estrus expression, silent estrus, ovulation rate, pregnancy outcome, and incidence of follicular cysts using Graph Pad Prism-6 software package (GraphPad Software Inc.). The value *p* < 0.05 was considered statistically significant.

## Results

### Experiment 1

In this experiment, buffaloes treated with GPG showed a higher estrus rate (96.1%; *P* < 0.05) with an increasing trend in ovulation (84.6%; *P* > 0.05), and pregnancy rate (30.8%; *P* > 0.05) with lower silent estrus (3.8%; *P* < 0.05) and follicular cyst (3.8%; *P* < 0.05) rates compared to PmPG, PGPE, and P_4_PmPG treated buffaloes. The diameter of the ovulatory follicle was similar in buffaloes treated with different protocols (GPG, PmPG, PGPE, and P_4_PmPG) (Table 1). In comparing the dynamics of follicle development and the size of follicles among four conventional methods, the GPG protocol was the best as rapid growth and the maximum diameter of follicles were observed (*P* < 0.05) at different time points compared to other treated groups, although the initiation of follicular development was started late compared to PmPG, and P4PmPG (Figure 3).

**Table 1:** Fertility parameters of crossbred buffaloes synchronized using PmPG, GPG, PGPE, and P_4_PmPG protocols during breeding season.

**Parameters**	**Synchronization protocols**			
	**PmPG (*n* = 28)**	**GPG (*n* = 26)**	**PGPE (*n* = 26)**	**P_**4**_PmPG (*n* = 14)**
Estrus rate (%)	23/28 (81.1)^ab^	25/26 (96.1)^a^	18/26 (69.2)^b^	11/14 (78.6)^ab^
Silent estrus (%)	5/28 (17.9)^ab^	1/26 (3.8)^a^	8/26 (30.8)^b^	3/14 (21.4)^ab^
Ovulation rate (%)	23/28 (82.1)	22/26 (84.6)	17/26 (65.4)	11/14 (78.6)
Pregnancy rate (%)	6/28 (21.4)	8/26 (30.8)	6/26 (23.1)	1/14 (7.1)
Follicular cysts (%)	5/28 (17.9)^ab^	1/26 (3.8)^b^	6/26 (30.8)^a^	3/14 (21.4)^ab^
Diameter of ovulatory follicle (mm)	13.7 ± 3.5	13.9 ± 3.4	13.3 ± 2.3	13.4 ± 2.2

*Values in brackets show the percentage of treated buffaloes for different studied variables. The superscripts in different small letters (a,b) in the same row indicate a statistical difference P < 0.05*.

### Experiment 2

In experiment 2, treatment of buffaloes with a dose of 0.4 mg/kg of mifepristone improved (*P* < 0.05) estrus expression, ovulation, and pregnancy rate, comparatively. A low (*P* < 0.05) number of buffaloes had follicular cysts and silent estrus when 0.4 mg/kg of mifepristone was combined in GPG protocol. In contrast, the ovulatory follicle size remained the same across the groups (Table 2). In addition, the follicular growth and size of follicles at different scanning times, depicts, comparatively, faster and larger in buffaloes received 0.4 mg/kg mifepristone (*P* < 0.05) on day 9 of GPG protocol compared to other dose groups (Figure 4).

**Table 2:** Effect of different doses of mifepristone (0, 0.3, 0.4, and 0.5 mg/kg) on estrus, ovulation, and pregnancy rate of buffaloes synchronized by GPGMH protocol during breeding season.

**Parameters**	**Mifepristone dose (mg/kg)**			
	**0 (*n* = 20)**	**0.3 (*n* = 17)**	**0.4 (*n* = 17)**	**0.5 (*n* = 17)**
Estrus response (%)	17/20 (85.0)^a^	13/17 (76.5)^ab^	15/17 (82.4)^a^	9/17 (52.9)^b^
Silent estrus (%)	3/20 (15.0)^b^	4/17 (23.5)^a^	2/17 (11.8)^b^	8/17 (47.1)^a^
Ovulation rate (%)	16/20 (80.0)^ab^	13/17 (76.5)^ab^	16/17 (94.1)^a^	9/17 (52.9)^b^
Pregnancy rate (%)	6/20 (30.0)^a^	4/17 (23.5)^b^	8/17 (47.1)^a^	6/17 (35.3)^a^
Follicular cysts (%)	1/20 (5.0)^a^	3/17 (17.6)^a^	1/17 (5.8)^a^	3/17 (17.6)^a^
Diameter of ovulatory follicle (mm)	12.4 ± 3.0	12.3 ± 2.1	14.3 ± 1.5	12.8 ± 2.4

*Values in brackets show percentage of treated buffaloes for different parameters. The superscripts in different small letters (a,b) in the same row indicate statistical difference P < 0.05*.

### Experiment 3

The buffaloes injected 0.4 mg/kg of mifepristone at the second GnRH in GPG protocol, showed a better (*P* < 0.05) pregnancy rate than buffaloes treated with mifepristone 4 h before or 4 h after the second GnRH of the GPG protocol. Estrus response, silent estrus rate, ovulation, and follicular cysts incidence remained the same across the groups (Table 3).

**Table 3:** Effect of mifepristone injection time (4 h before, same time, or 4 h after second GnRH) on estrus, ovulation, and pregnancy of buffaloes synchronized by GPGMH during breeding season.

**Parameters**	**Time injection of mifepristone**		
	**4 h Before (*n* = 18)**	**Same time (*n* = 17)**	**4 h After (*n* = 17)**
Estrus response (%)	16/18 (88.9)	17 (100)	16/17 (94.1)
Silent estrus (%)	2/18 (1.1)	0/17 (0)	1/17 (5.9)
Ovulation rate (%)	16/18 (88.9)	16/17 (94.1)	14/17 (82.4)
Pregnancy rate (%)	2/18 (11.1)^b^	6/17 (35.3)^a^	2/17 (11.8)^b^
Follicular cysts (%)	2/18 (11.1)	0/17 (0)	1/17 (5.9)

*Values in brackets show the percentage of treated buffaloes for studied parameters. The superscripts in different small letters (a,b) in the same row indicate a statistical difference P < 0.05*.

### Experiment 4

In this experiment, the timing of single AI (20, 24, or 28 h) following mifepristone or second GnRH did not influence the pregnancy outcomes. However, when AI was performed at 24 h after mifepristone or second GnRH in GPGMH treated buffaloes, they showed a higher (*P* > 0.05) pregnancy rate numerically. The GPGMH treated buffaloes, whether inseminated twice at 20 and 28 h or 16 and 26 h, did not show any variability in pregnancy rates (Table 4.)

**Table 4:** Fertility parameters of buffaloes synchronized by GPGMH protocol in connection to different AI times and frequency.

	**AI time (after mifepristone injection)**				
**Parameters**	**Single AI (h)**			**Double AI (h)**	
	**20 (*n* = 16)**	**24 (*n* = 16)**	**28 (*n* = 17)**	**20 and 28 (*n* = 25)**	**16 and 26 (*n* = 26)**
Estrus response (%)	14/16 (87.5)	15/16 (93.8)	13/17 (76.5)	20/25 (80.0)	21/25 (80.7)
Silent estrus (%)	2/16 (12.5)	1/16 (6.3)	4/17 (23.5)	5/25 (20.0)	4/25 (15.4)
Ovulation rate (%)	14/16 (87.5)	15/16 (93.8)	13/17 (76.5)	20/25 (80.0)	21/25 (80.7)
Pregnancy rate (%)	5/16 (31.2)	7/16 (43.8)	6/17 (35.5)	6/25 (24.0)	10/25 (38.5)
Follicular cysts (%)	2/16 (12.5)	0/16 (0)	1/17 (5.9)	2/25 (8.0)	1/25 (3.8)

### Experiment 5

In this experiment, there was no significant difference in estrus expression, ovulation rate, ovulatory follicle diameter, and pregnancy outcome in GPGMH, P_4_GPG, and GPG treated buffaloes. However, the incidence of silent estrus (*P* > 0.05) and follicular cyst (*P* < 0.05) was comparatively lower in GPGMH treated buffaloes than P_4_GPG and GPG treated buffaloes (Table 5).

**Table 5:** Comparison of estrus, ovulation, and pregnancy rate of buffaloes synchronized by GPG, GPGMH, and P_4_GPG protocols during breeding season.

**Parameters**	**FTAI synchronization protocol**		
	**GPGMH (*n* = 45)**	**P4GPG (*n* = 41)**	**GPG (*n* = 30)**
Estrus response (%)	42/45 (93.3)	33/41 (80.5)	26/30 (86.7)
Silent estrus (%)	3/45 (6.7)	8/41 (19.5)	4/30 (13.3)
Ovulation rate (%)	41/45 (91.1)	33/41 (80.5)	23/30 (76.7)
Pregnancy rate (%)	19/45 (42.2)	14/41 (34.1)	10/30 (33.3)
Follicular cysts (%)	1/45 (4.4)^b^	10/41 (24.4)^a^	3/30 (10.0)^ab^
Diameter of ovulatory follicle	13.1 ± 2.5	12.3 ± 3.1	13.4 ± 3.0

*Values in brackets show the percentage of treated buffaloes for different parameters. The superscripts in different small letters (a,b) in the same row indicate a statistical difference P < 0.05*.

## Discussion

Reduced reproductive performance is one of the major reasons for low profitability in the buffalo industry, especially for crossbred buffaloes. Several protocols and strategies of estrus synchronization and FTAI have been previously reported in different buffalo breeds (7, 9, 10). The most commonly used estrus synchronization and FTAI protocol in lactating buffaloes is the Ovsynch program (11), previously named GPG. Previous studies showed that the GPG program treatment in cycling buffaloes during breeding season resulted in variable pregnancy rates from 30 to 60% (8, 15). The use of the Ovsynch program of cyclic river-type crossbred buffaloes (Murrah x Mediterranean) resulted in high conception rates of 56% during the breeding season (12). In the present study, the crossbred buffaloes treated with GPG protocol showed better estrus (96%) and conception rate (31%) than other conventional protocols. Although the conception rate in the present study was much lower, as reported in river-type buffaloes (12), it might be linked to breeding differences, management, and the environmental conditions of study sites. Follicular development was rapid, because of development in GPG protocol that corroborates earlier studies (17, 18). The increased follicular development in this study was linked to the selection of animals with similar follicular stages at the first injection of GnRH in GPG protocol. However, the correlations of hormonal patterns to follicular dynamics are lacking in this study.

One of the major associated problem of GPG, as previously reported (19), is a higher incidence of follicular cysts in buffaloes, especially when treated for several cycles (13). According to an earlier report, the follicular cyst rate in buffalo varies from 6 to 19% after GPG treatment (20). It has been postulated that high P_4_ concentrations possibly inhibit the onset of LH surge following the second GnRH, leading to the formation of follicular cysts (13). The inclusion of mifepristone, as an anti-progestogen by blocking the inhibitory effects of P_4_ on LH surge, (9, 21) in GPG based protocol, was tested to minimize the incidence of follicular cysts in crossbred buffaloes. The results showed that the inclusion of mifepristone (0.4 mg/kg) in GPG based protocol significantly reduced the incidence of follicular cysts (2.1%) in buffaloes compared to conventional GPG based protocol (6.6%). The use of mifepristone was advantageous for follicular development, whereas, a medium dose of mifepristone (0.4 mg/kg) promoted the ovulatory follicle size. This comparison indicates that the inclusion of mifepristone successfully declined the P_4_ level in crossbred buffaloes for better application of GPG protocol. However, a comparison of LH and progesterone following mifepristone injection in GPGMH based protocol could explain the mechanisms and application better in buffaloes.

Silent estrus is also one of the major problem in buffaloes when estrus detection is limited and leads to increased calving intervals (21). It has been reported that the silent estrus rate in buffalo increased up to 38% even during the breeding season (22). PGF_2_α or its analog induces luteolysis with a marked decline in P_4_ (23), whereas, an inappropriate decline in plasma P_4_ concentration and the CL area after PGF_2_α treatment in GPG protocol could be a possible reason for silent estrus behavior. Therefore, the inclusion of mifepristone in the GPG protocol seems to be a better strategy for reducing the silent estrus in crossbred buffaloes. The present study showed that buffaloes treated with 0.4 mg/kg mifepristone combined with the GPG regimen during the breeding season, resulted in a lower silent estrus rate (6%) compared with GPG treatment (11%).

The present study showed that ovulation in GPG protocol was not highly synchronized, hence, it resulted in a relatively low pregnancy rate. Currently, multiple synchronization protocols are used with a combination of exogenous hormones to regulate the estrous cycle (9, 24). The circulating P_4_ concentration influences the LH surge release; therefore, the decreasing trend of serum P_4_ concentration during the follicular growth phase until ovulation is requisite for ovulation occurrence (25). Earlier studies have reported that there is an ~80% ovulation rate in buffaloes when treated through conventional GPG treatment during the breeding season (26). On the other hand, the current results showed that GPG treatment during the breeding season resulted in similar ovulation rates (80%), but the inclusion of (0.4 mg/kg) mifepristone in GPG enhanced the ovulation rate significantly (93%).

FTAI has been adopted to overcome the difficulty of estrus detection and apply AI in spontaneously ovulating animals at a predetermined time (6, 27). Studies on Murrah buffaloes showed that the ovulation occurred at 23.2 ± 1.0 h of the second GnRH injection (15) and 26.5 and 24.4 h after the second GnRH or LH when GPG protocol was applied in crossbred (Murrah × Mediterranean) buffaloes, respectively (11). The Ovsynch synchronization program with AI at 20 h after the second GnRH in Mediterranean river buffaloes resulted in a 48% pregnancy rate during the breeding season (28). Some other studies have shown a similar pregnancy rate (45%) when AI was performed 16 h after the second GnRH injection during the breeding season (29). In the case of cows, single AI is recommended in Ovsynch protocol because of more predictable tight ovulation synchrony (30). However, pregnancy rates were 38 and 33% in Thai swamp (31) and Murrah (8) buffaloes when inseminated at two fixed times of 12 and 24 h after the second GnRH in Ovsynch synchronization protocol during the breeding season. In the present study, a higher pregnancy rate (44%) was obtained in buffaloes when FTAI was carried out at 24 h after mifepristone of the second GnRH injection of GPGMH protocol during the breeding season.

Synchronization of P_4_, CIDR along with GnRH has shown promising results in terms of pregnancy in buffalo (24), particularly during summer anestrous (32). Other studies have reported that higher P_4_ concentrations in FTAI protocols could compromise follicular development, ovulation, and pregnancy rates (33). The combination of CIDR and insulin in modified Ovsynch TAI programs resulted in a 46% pregnancy rate in buffaloes during the breeding season (34, 35). In the present study, modified Ovsynch TAI protocol (GPGMH) reduced the incidence of silent estrus rate (7%) and follicle cysts rate (0%) with increased estrus response (93%), ovulation (91%), and pregnancy rate (42%) in comparison to P_4_GPG or conventional GPG regimens during the breeding season.

## Conclusion

The current study supported the new modified of GPG protocol, (GPGMH; Figure 2) by the inclusion of mifepristone (with a dose of 0.4 mg/kg along the second GnRH), AI after 24 h of mifepristone or second GnRH, and post-AI hCG (at day 5 of AI), is a choice regimen to enhance ovulation and pregnancy rates by reducing the incidence of follicular cysts and silent estrus in crossbred buffaloes during the breeding season.

## Data Availability Statement

The original contributions presented in the study are included in the article/supplementary material, further inquiries can be directed to the corresponding author/s.

## Ethics Statement

The study was reviewed and approved by The Ethical Committee of the Hubei Research Center of Experimental Animals (Approval ID: SCXK (Hubei) 20080005). Written consent was obtained from the owners for the use of animals in this study.

## Author Contributions

All authors listed have made a substantial, direct and intellectual contribution to the work, and approved it for publication.

## Conflict of Interest

The authors declare that the research was conducted in the absence of any commercial or financial relationships that could be construed as a potential conflict of interest.

## Publisher's Note

All claims expressed in this article are solely those of the authors and do not necessarily represent those of their affiliated organizations, or those of the publisher, the editors and the reviewers. Any product that may be evaluated in this article, or claim that may be made by its manufacturer, is not guaranteed or endorsed by the publisher.
